# Dosimetric and spectroscopic study of LiMgPO_4_ doped with Tm^3+^ and Er^3+^

**DOI:** 10.1039/d2ra07109f

**Published:** 2023-02-08

**Authors:** Jingyuan Guo, Chenxi Jian, Caixing Zeng, Zhengye Xiong, Luyan Wang, Dongcui Zhou

**Affiliations:** a School of Electronic and Information Engineering, Guangdong Ocean University Zhanjiang 524088 China xiongzhengye@139.com; b Faculty of Chemistry and Environment Science, Guangdong Ocean University Zhanjiang 524088 China

## Abstract

Olivine-type phosphate LiMgPO_4_ doped with rare earth elements is considered a novel dosimetric material with excellent performance that is suitable for thermoluminescence (TL) and optically stimulated luminescence (OSL) measurements. Novel LiMgPO_4_:Tm,Er samples were synthesized by a high-temperature solid-state reaction method. A detailed study of the TL and OSL of the samples was performed using β-ray irradiation and X-ray-excited optical luminescence (XEOL) spectroscopy. Density functional theory (DFT) calculations were performed to predict the preferential positions of thulium and erbium, and photoluminescence (PL) spectra and TL 3D spectra were analyzed. The DFT calculation results show that Mg is preferentially replaced by Tm/Er in the LiMgPO_4_ system. The PL/TL3D/XEOL spectra of the samples are dominated by the characteristic luminescence of Tm^3+^, and the OSL decay curve of photoluminescence has fast and slow decay components with decay constants of 5 s and 42 s, respectively. The TL and XEOL results show that LiMgPO_4_:Tm,Er has strong emission signals under different types of radiation rays. The PL/TL3D/XEOL spectral results show that Er^3+^ has no radiative excitation, but Tm^3+^ has strong luminescence, such that the sample still emits strong TL and PL signals. Two TL emission peaks occur at approximately 120 °C and 300 °C, where the high-temperature peak is significantly more intense than the low-temperature peak, promoting the stability of the TL and OSL signals of the samples. The TL curve consists of 6 general TL dynamic peaks. The nonlinear parameters of the TL dose response are *R* = 0.08 and *D*_0_ = 479 Gy, and the OSL dose response is linear in the range of 0.2–1000 Gy. The TL and OSL signals of the LiMgPO_4_:Tm,Er phosphor have good repeatability. Therefore, the LiMgPO_4_:Tm,Er phosphor can be used for radiation dose measurement.

## Abstract 2

A novel olivine type phosphate dosimetric material doped with Tm and Er with excellent TL and OSL properties.

## Graphical abstract

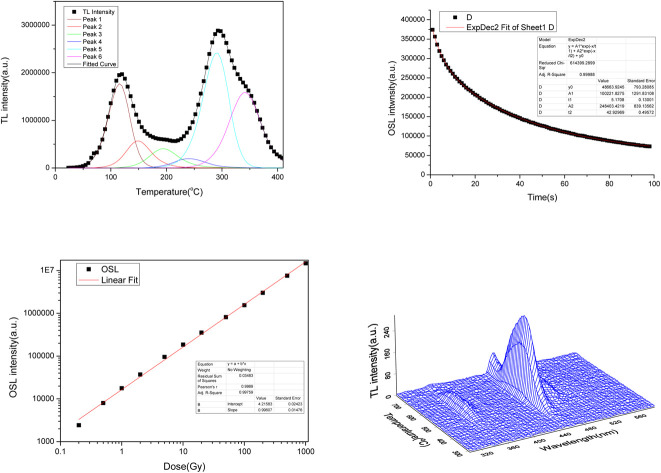

## Introduction

Ionizing radiation is widely used in life. Apart from industrial, scientific and nuclear applications, ionizing radiation is increasingly used in medical diagnosis and cancer treatment. Radiation measurement methods need to be developed to ensure maximum radiation safety. Thermoluminescence (TL) and optical stimulated luminescence (OSL) play an important role in luminescence dosimetry and have been widely used for radiation dose measurement, dating, environmental monitoring, geology and other fields. Both dosimetry methods involve the use of thermal/photoexcitation to monitor the radiation dose in the laboratory after radiotherapy. X-ray-excited optical luminescence (XEOL) occurs spontaneously and immediately upon exposing a sample to ionizing radiation, where the XEOL strength is proportional to the dose rate. Currently, TL and OSL phosphors suitable for dosimetry mainly include LiF:Mg,Ti,^1^ CaF_2_:Mn,^2^ Al_2_O_3_:C,^3–5^ CaSO_4_:Dy,^6^ SrSO_4_:Eu,^7^ and Li_2_B_4_O_7_:Mn.^8^ Al_2_O_3_:C is one of the most widely used dosimetry materials. Research on real-time dosimeter systems mainly focuses on commercial Al_2_O_3_:C^9–11^ and BeO^12^ that are used as detectors. In addition to luminators, doped silica fibres have been successfully used in RL detectors in remote measurement systems.^13,14^

Many efforts have been made to develop novel dosimetry materials with excellent performance. Lithium magnesium phosphate (LiMgPO_4_, LMP) is a relatively novel and promising dosimetric material that has been extensively studied. The doped LMP has both a strong TL and a significant OSL^15–17^ and can therefore be used as both a TL dosimeter and an OSL dosimeter.

Zhang *et al.*^18^ studied the luminescence characteristics of LMP:Eu and the influence of the doping concentration on the LMP:Eu crystal structure. Menon *et al.*^19^ developed a LMP:Tb phosphorescent as a novel dosimetric material suitable for TL and OSL applications. This material is competitive with those used in mainstream commercial detectors. The TL signal sensitivity of LMP:Tb is 2.5 times that of CaSO_4_:Dy. However, the temperature for the main emission peak (approximately 170 °C) is too low to maintain peak stability, and the TL signal intensity declines by 7% in one month at room temperature. The LMP:Tb,B phosphor is an OSL material with good performance.^20–23^

Dhabekar *et al.*^24^ reported that the main TL peak temperature of LMP:Tb,B is 230 °C. The OSL intensity of LMP:Tb,B under excitation of by 470 nm blue light is 1.3 times higher than that of Al_2_O_3_:C. The excitation and emission spectra of LMP:Tb,B do not overlap, and the production process of LMP:Tb,B is simple. The linear range of LMP:Tb,B is 1 mGy to 1 kGy. LMP:Tb,B exhibits high TL and OSL efficiencies under irradiation by γ or X-rays, as well as UV, photons, protons, neutrons, α particles^25^ and β sources.^26^

Various doped LMP phosphors have also been developed. Gai *et al.*^27^ found that adding Sm^3+^ to LMP:Tb,B phosphors increases the OSL sensitivity by approximately one-fold, resulting in a linear dose response range of 0.1–216 Gy.

Gai *et al.* also studied another LMP phosphor, LMP:Eu,Sm,B, which has a main-luminescence-peak temperature of 354 °C and can be used for real-time dosimetry.^28^

Kellerman *et al.*^29^ synthesized LMP:Er samples by a solid-state reaction method. The X-ray luminescence, TL and OSL of the samples was studied. Density functional theory (DFT) calculations were performed on doped structures, and the preferred positions of charge compensation vacancies and Er^3+^ were predicted. The dynamics of LMP in the form of powder,^30–34^ crystals^35–39^ and a thin foil was studied.^40,41^

To further explore the TL and OSL properties of LMP, the luminescence kinetic parameters of undoped crystals were extensively studied.^42^ Considerable research^43–47^ has also been conducted on defects in prepared and irradiated lithium magnesium phosphate. Kellerman *et al.*^48^ synthesized LiMgPO_4_:RE (Re-nd, Sm, Gd, Tb, Dy, Ho, Er, Tm) phosphate by conventional solid phase reaction method, and studied its optical properties by experimental and theoretical methods. Miao *et al.*^49^ designed and developed a promising wideband SWIR phosphor by introducing Cr^3+^–Ni^2+^ ion pair into LiMgPO_4_ host. Under 450 nm blue excitation, the synthesized LiMgPO_4_:Cr^3+^, Ni^2+^ phosphors exhibit extensive and intense SWIR emission in the range of 1100–1600 nm, with a peak wavelength of 1380 nm and a half-width of 273 nm, due to the effective energy transfer from Cr^3+^ to Ni^2+^.

LMP has high sensitivity to ionizing radiation, good repeatability, a linear dose response and tissue equivalence. A large number of research results have demonstrated that materials with an LMP matrix are candidate materials for real-time detectors used in applications such as medical monitoring, environmental dosimetry and space dosimetry. In this study, LMP:Tm,Er phosphors were prepared by a high-temperature solid-state method, and the PL, TL, OSL and XEOL of the samples were investigated in detail. The formation energies of Tm and Er were determined using *ab initio* calculations, and the preferential positions of Tm and Er were predicted.

## Experimental

Experiments were performed using raw materials of LiOH·H_2_O, NH_4_H_2_PO_4_ and Mg(NO_3_)_2_·6H_2_O, all of which were analytically pure. The rare earth compounds used were Tm_2_O_3_ and Er_2_O_3_ at a purity of 99.9%. The doping concentrations of Tm^3+^ and Er^3+^ were 0.5 mol% and 0.02 mol%, respectively. Samples were synthesized using a high-temperature solid-state reaction method. The main steps used to prepare the materials are given below.

(1) We placed appropriate quantities of LiOH·H_2_O, NH_4_H_2_PO_4_, Mg(NO_3_)_2_·6H_2_O and rare earth impurities into an agate mortar and ground these materials with deionized water into a uniform suspension.

(2) The resulting samples were evaporated to dryness. The resulting paste was transferred to alumina cups placed on an electric hot plate to yield a dry sample powder.

(3) The filled alumina cups were placed in a tube furnace and heated for 2 h at 1000 °C. The alumina cups were then removed from the furnace and cooled to room temperature. The samples were ground to powders, which were stored in a sample bag.

TL/OSL glow curves were measured using an automatic Risø TL/OSL-15-B/C reader system equipped with a ^90^Sr beta source and a U-340 detection filter. The OSL readouts were obtained using blue light emitting diodes (LEDs) with a peak emission at 470 nm and a power of 50 mW cm^−2^, where the OSL signals were recorded for 100 s. The heating rate for TL measurement and preheating was set to 5 °C s^−1^. The radiation dose rate was approximately 0.1 Gy s^−1^. The sample was irradiated for a prescribed time period *t* (1–20 000 s) and then heated linearly from room temperature to 500 °C, and the TL signals were recorded.

The structures of the LiMgPO_4_ samples were analysed using a D-MAX 2200 VPC X-ray diffractometer (XRD) with Cu radiation in a 2*θ* range of 10°–80° using a velocity of 5° min^−1^.

The photoluminescence (PL) spectra of the phosphor were measured with a HitachiF-4500 fluorescence spectrometer. A xenon lamp was used as the excitation light source.

TL 3D and XEOL spectra of the synthesized samples were measured using an LTTL3DS spectrometer (Guangzhou Radiation Science and Technology Co. Ltd). These spectra supplemented the TL glow curve with wavelength-related information related to TL traps and defect structures.^50,51^ The temperature of the samples during the TL3D and XEOL spectral measurement was increased linearly at a rate of 2 K s^−1^. To measure the TL3D spectrum, a sample was placed in a sample tank and irradiated with X-rays. The TL3D spectrum of the sample was then measured. To measure the XEOL spectra, a sample was also placed in a closed sample tank, vacuumed with a vacuum pump (∼1 Pa), and cooled to approximately 80 K by liquid nitrogen. Then, XEOL spectra were measured at different temperatures while the sample was irradiated by X-rays. The X-ray tube was operated at a voltage of 50 kV and a current of 150 μA. The current was adjusted to maintain the sample temperature between 80 K and 800 K. The wavelength ranged from 200 to 850 nm.

The LMP crystal has an olivine structure and cell parameters of *a* = 0.4739 nm, *b* = 0.5972 nm, *c* = 1.0255 nm, and *α* = *β* = *γ* = 90°. A cell model for a perfect LiMgPO_4_ crystal with 28 atoms was established, where each cell was composed of 4 LMP units. The first-principles software Vienna *ab initio* Simulation Package (VASP) based on density functional theory was used to optimize the structure of the LMP cells, and VESTA software was used to build the model and read the lattice parameters of the LMP cells. Exchange and correlation potentials were described by the generalized-gradient approximation (GGA) with the Perdew–Becke–Ernzerhof functional.^52^ A convergence test was performed, and plane waves with a kinetic energy cut-off of 650 eV were set as the basis set. The convergence criterion of the total energy of the system was 1 × 10^−6^ eV per atom, and the force convergence criterion was 1 × 10^−2^ eV A^−1^. The gamma method was used to conduct grid sampling of *k* points in the Brillouin region. A 4 × 3 × 2 grid-point segmentation scheme was used to perform a self-consistent calculation of the number of *k* grids (KPOINTS). The conjugate gradient optimization algorithm was used to minimize the electron energy, and the parameter ISIF = 3 was set for structural optimization, that is, the volume and ion positions of the lattice were optimized simultaneously. We first optimized the structure of the LMP single crystal cell and then calculated the doping formation energy of Li_1−*x*_Tm_*x*_MgPO_4_, LiMg_1−*x*_Tm_*x*_PO_4_ and Li_1−*x*_Er_*x*_MgPO_4_, LiMg_1−*x*_Er_*x*_PO_4_ (*x* = 0.125, 0.25, and 0.33) under three doping concentrations: *x* = 0.125 (2 × 2 × 2 supercell 224 atoms), *x* = 0.25 (1 × 2 × 2 supercell 112 atoms), and *x* = 0.33 (1 × 1 × 3 supercell 84 atoms). Finally, the optimal structure of the doping system was obtained.

## Results and discussion

### X-ray diffraction (XRD) analysis

**Fig. 1:**
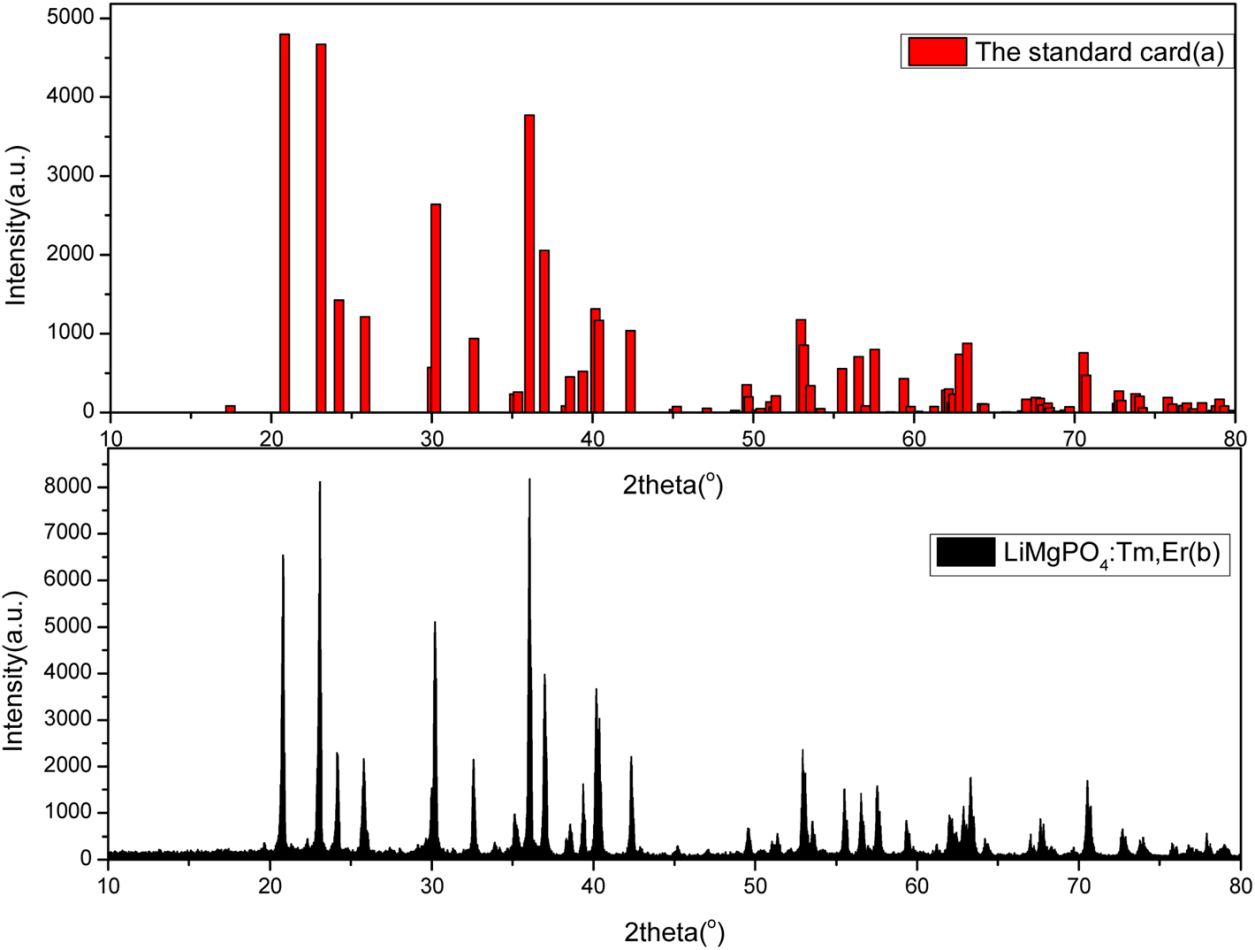
Comparison of the XRD pattern of the LiMgPO_4_:Tm,Er sample (b) and the standard XRD card (no. 00-032-0574) (a).

Fig. 1(a) shows the standard card of LMP, which belongs to the orthorhombic system with lattice constants of *a* = 1.0147 nm, *b* = 0.5909 nm, and *c* = 0.4692 nm; Fig. 1(b) shows the XRD patterns of the LMP:Tm,Er samples. The number and location of the XRD peaks indicate that the sample had the same crystal structure as undoped LMP. The incorporation of doping impurities into the sample did not significantly change the spectrum. That is, neither the number nor the location of the peaks differed between the spectra of the doped and undoped samples. The strong peaks in the spectrum of the doped sample were in good agreement with those in the spectrum of the undoped sample, indicating that the prepared LMP had a high purity, the doping impurities had almost no effect on the sample purity, and the crystal structure did not change with doping. Compared with the XRD spectrum of Er_2_O_3_, no characteristic diffraction peak of Er_2_O_3_ was found in the XRD spectrum of the LMP sample, confirming that Er ions were indeed doped into the lattice of the matrix.

### DFT

We calculated the substitution formation energy of Li and Mg for doping Tm and Er into LMP, where the substitution formation energy *E*_f_ (Tm→Li) was calculated as follows:

1 *E*_f_(Tm→Li) = *E*_tot_(Li_1−*x*_Tm_*x*_MgPO_4_) + *μ*Li − *E*_tot_(LiMgPO_4_) − *μ*Tm

and the substitution formation energy *E*_f_(Er→Li) was calculated using the following formula:

2 *E*_f_(Er→Li) = *E*_tot_(Li_1−*x*_Er_*x*_MgPO_4_) + *μ*Li−*E*_tot_(LiMgPO_4_) − *μ*Er

Formulas for the formation energy for the replacement of Mg by Tm and Er were obtained in the same way. In the formula presented above, *E*_tot_ (LiMgPO_4_), *E*_tot_ (Li_1−*x*_Tm_*x*_MgPO_4_) and *E*_tot_ (Li_1−*x*_Er_*x*_MgPO_4_) are the total energies of pure LMP after structural optimization, Li substitution by doping with Tm, and Li substitution by doping with Er, respectively. *μ*Li, *μ*Tm and *μ*Mg, *μ*Er are the chemical potentials of the Li, Tm, Mg, Er atoms, respectively, that were used to calculate the energy of an Li, Tm, Mg, or Er atom in the bulk structure, respectively.

Table 1 shows that the doping formation energy changes little with increasing concentration. The doping formation energy is smaller for substituting Mg with Tm/Er than for substituting Li with Tm/Er at the three considered doping concentrations. The lower the doping formation energy is, the more stable the structure is. Therefore, Tm/Er doped into the LMP system is predicted to preferentially substitute Mg.PL

**Table 1:** Doping formation energy (eV) of various doping systems

Supercell	1 × 1 × 3 (33%)	1 × 2 × 2 (25%)	2 × 2 × 2 (12.5%)
LMP Tm–Li	4.3313	4.3435	4.7048
LMP Tm–Mg	2.2666	2.2735	2.4168
LMP Er–Li	4.0173	3.9438	4.0537
LMP Er–Mg	1.7368	1.6396	1.821

**Fig. 2:**
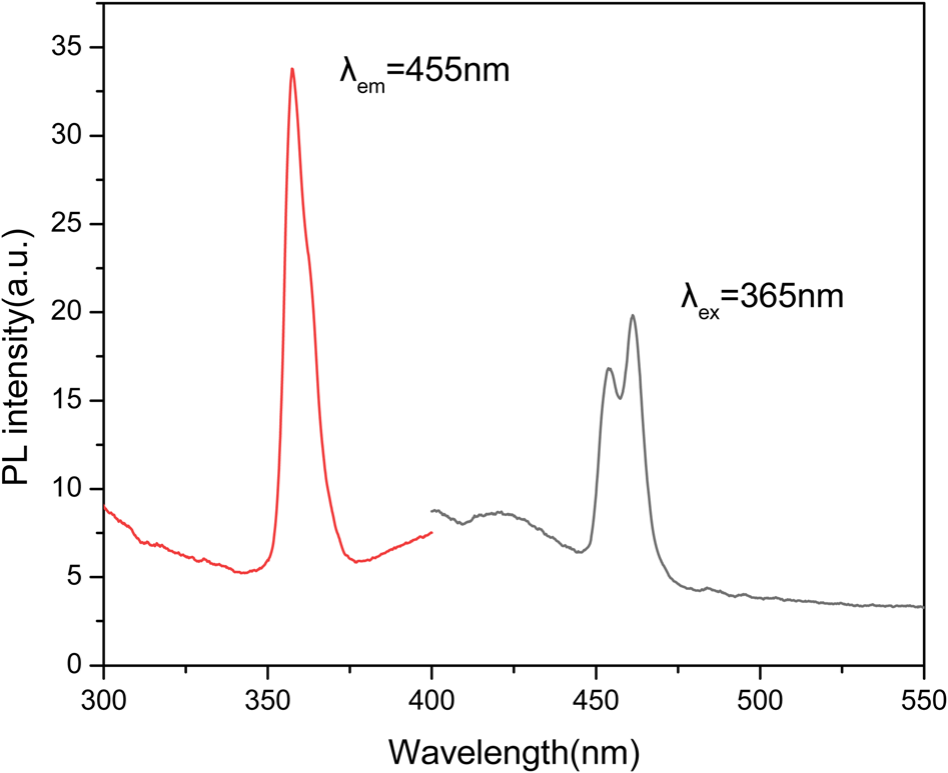
PL spectra of the LiMgPO_4_:Tm,Er phosphor.

The PL spectra of the LMP:Tm,Er phosphor at room temperature are shown in Fig. 2. The emission spectrum obtained by exciting the phosphor at 365 nm contains 2 peaks at 455 and 465 nm. The emission wavelengths of 455 and 465 nm correspond to the characteristic luminescence of Tm^3+^ ions. The PL spectra of LMP:Tm,Er do not contain peaks produced by 4f–4f transitions in erbium. This result is similar to that obtained for LMP: Er.^53^

### TL3D spectra

**Fig. 3:**
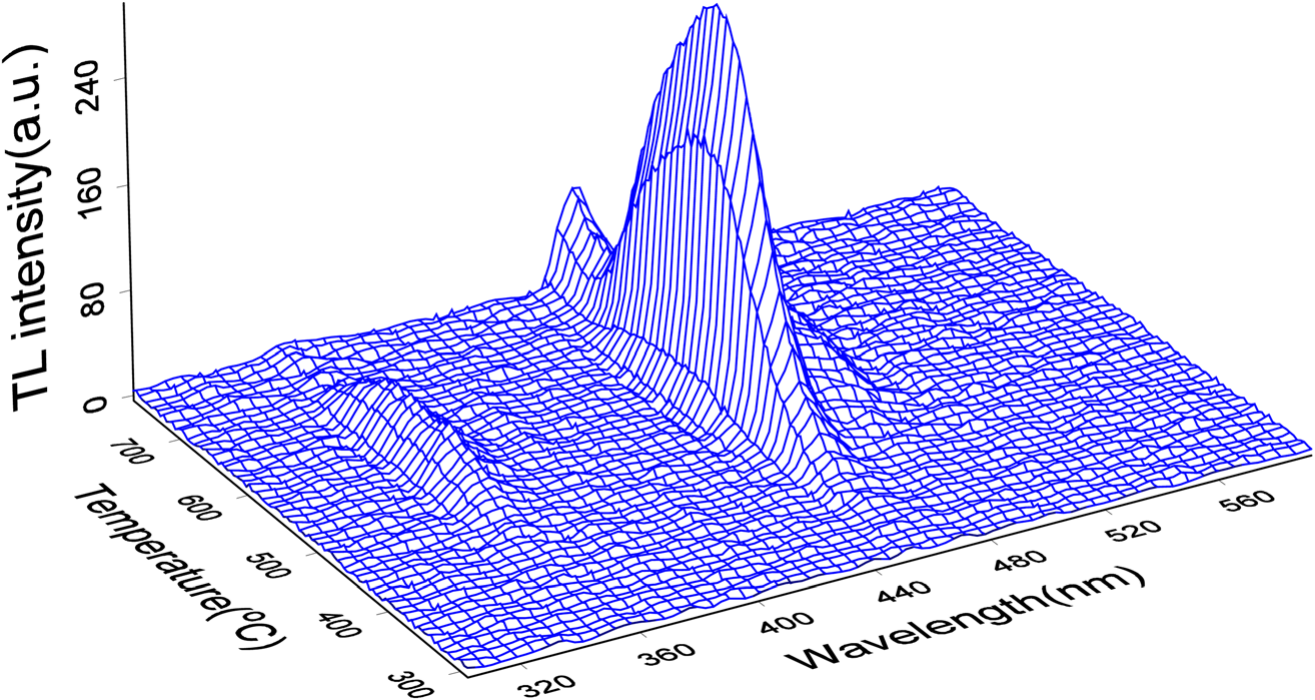
TL 3D spectra of LMP:Tm,Er.

The TL3D spectra of LMP:Tm,Er irradiated with X-rays at 100 Gy are shown in Fig. 3. The sample codoped with Tm^3+^ and Er^3+^ has a luminescence peak temperature of 573 K, and the main luminescence wavelength is 455 nm, corresponding to the characteristic luminescence of Tm^3+^.^54^ The main TL emission wavelengths are 362 nm and 455 nm, corresponding to the luminescence of the ^1^D_2_ → ^3^H_6_, ^1^D_2_ → ^3^H_4_ transition of Tm^3+^, for which the luminescence is most intense at 455 nm and the peak temperature for the main luminescence peaks is 558 K. The Er^3+^ transition is an f–f transition. The effective shielding of the 4f electrons from the surrounding environment by the 5s^2^ and 5p^6^ shells only causes a slight change in the crystal field of the matrix material. Therefore, this shielding appears as a narrow band. The wavelength of the luminescence in the TL 3D emission spectrum is identical to that observed for PL. This result indicates that the luminescence wavelength of the rare-earth-ion-doped phosphor material is mainly determined by the transition between the energy levels of the incorporated rare earth element. The main glow wavelengths of LMP:Tm and Er match well with the spectral response of the photomultiplier tube, which enhances the measurement efficiency of the TL glow curve.

### XEOL

**Fig. 4:**
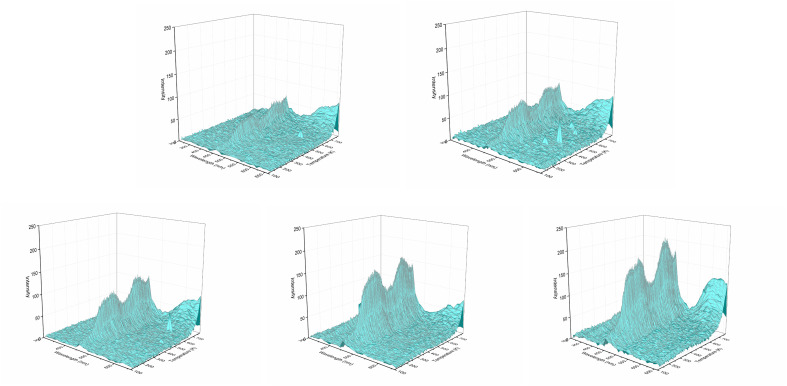
XEOL spectra of LMP:Tm,Er phosphors with different X-ray output power (the X-ray tube has a constant voltage of 50 kV. From left to right, the current in the first row is 50, 80 μA, and the current in the second row is 100, 150, 180 μA).

**Fig. 5:**
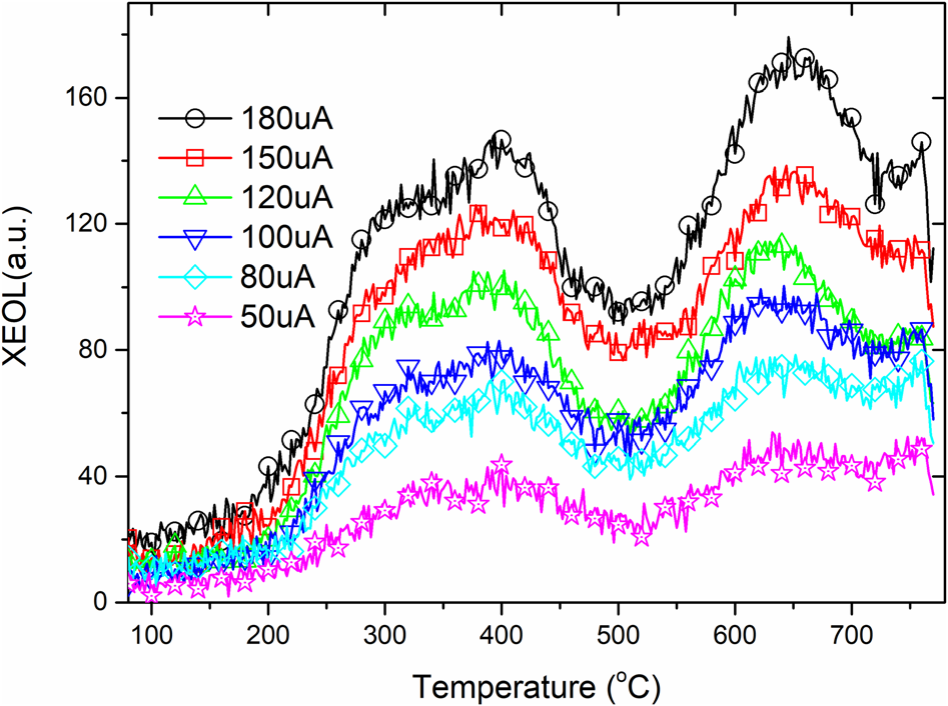
XEOL of LMP:Tm,Er phosphor at different temperature and X-ray output power.

**Fig. 6:**
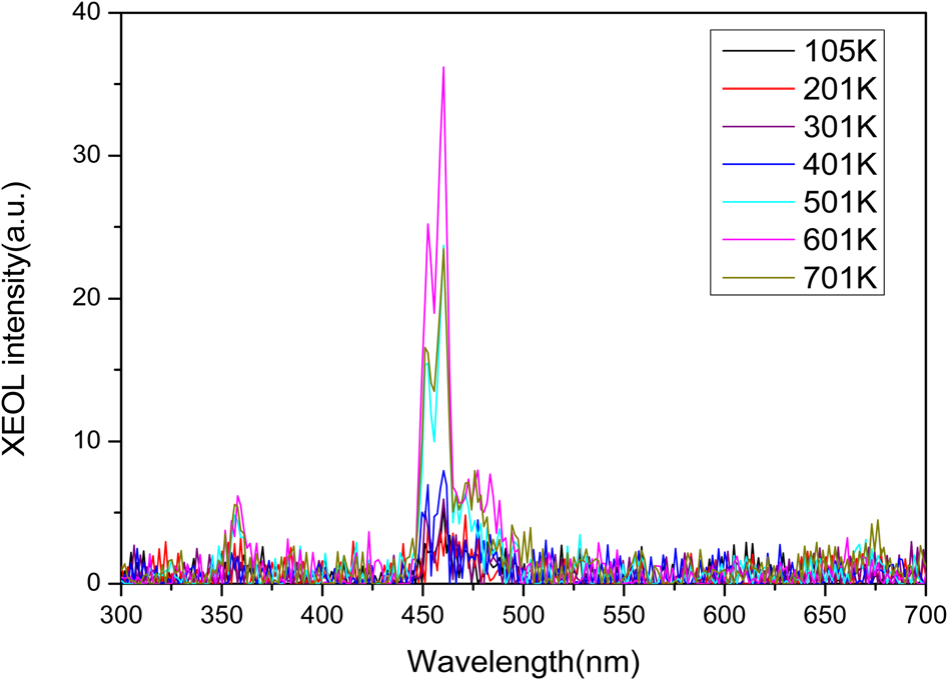
XEOL spectra of LMP:Tm,Er phosphors at different temperatures.

Rare earth ions in crystals produce luminous emission bands during X-ray excitation. As X-ray radiation penetrates the entire material, XEOL spectra provide fairly detailed spectral data.^55,56^Fig. 4 and Fig. 5 shows the XEOL spectra of LMP:Tm,Er phosphors at different temperature and X-ray output power. It can be seen from Fig. 4 and 5 that the luminescence intensity of the sample increases with the increase of the X-ray tube output power. Fig. 6 shows the XEOL spectra obtained by irradiating the LMP:Tm,Er phosphor with X-rays (∼0.1 Gy s^−1^) at different temperatures. Two important emission bands appear in the XEOL spectra, a purple emission band in the range of 350–375 nm and a blue emission band in the range of 450–500 nm. The peak locations for the XEOL spectra are almost identical to those for the PL spectra (Fig. 2) and TL3D spectra (Fig. 3). Fig. 6 shows that the XEOL luminescence intensity increases with the temperature for 105–601 K but is lower at 701 K than at 601 K. This result shows that the sample has good XEOL performance and good thermal stability at high temperature. For a fixed radiation intensity, the luminescence intensity increases with the temperature. This phenomenon is caused by the conversion of the energy stored in the internal defects of phosphors into light radiation energy.^57^ Increasing the temperature (105–601 K) results in thermal broadening in the XEOL spectra; that is, the half-width of the luminescent peak increases with the temperature. The half-width of the XEOL luminescent peak above 701 K is lower than that at 601 K. The results are in accordance with the ref. 58. The change of fluorescence intensity of rare earth doped upconversion nanoparticles with temperature conforms to the Boltzmann distribution.

### TL and OSL

**Fig. 7:**
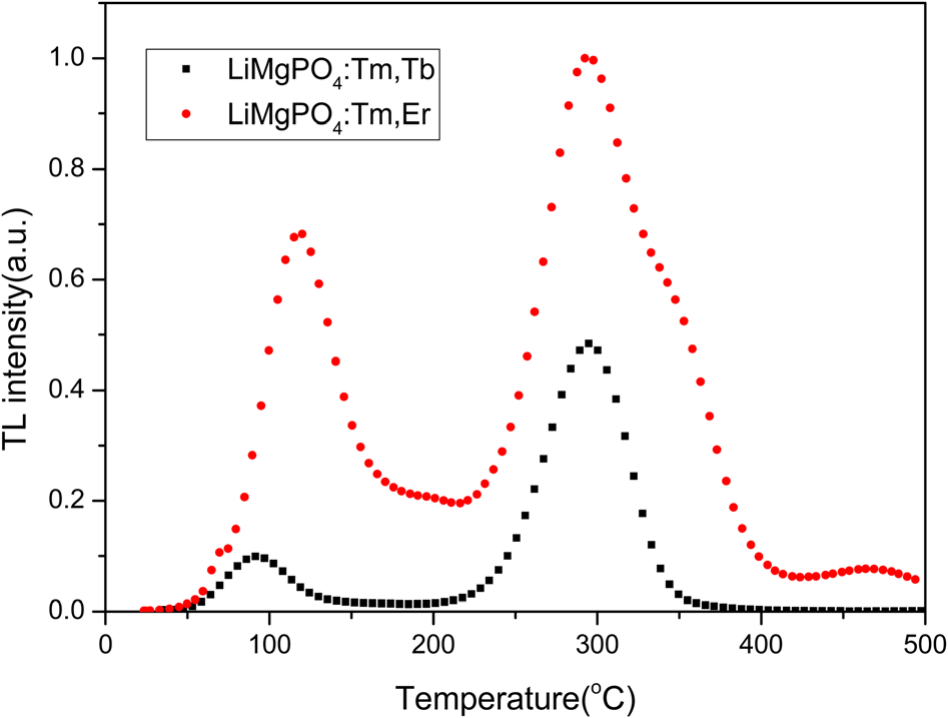
Comparison of the TL intensities of LiMgPO_4_:Tm,Er and LiMgPO_4_:Tm,Tb.

The phosphors LMP:Tm,Er and LMP:Tm,Tb have equal masses when exposed to the same dose (1 Gy) from a ^90^Sr beta source. Fig. 7 compares the TL glow curves of LMP:Tm,Er and LMP:Tm,Tb obtained under the same measurement conditions. The TL integral intensities of the two samples were compared. The TL intensity of the LMP:Tm,Er phosphor is higher than that of LMP:Tm,Tb. As LMP:Tm,Tb has a similar TL sensitivity to Al_2_O_3_:C,^59^ LMP:Tm,Er can also be used for low-dose measurements.

**Fig. 8:**
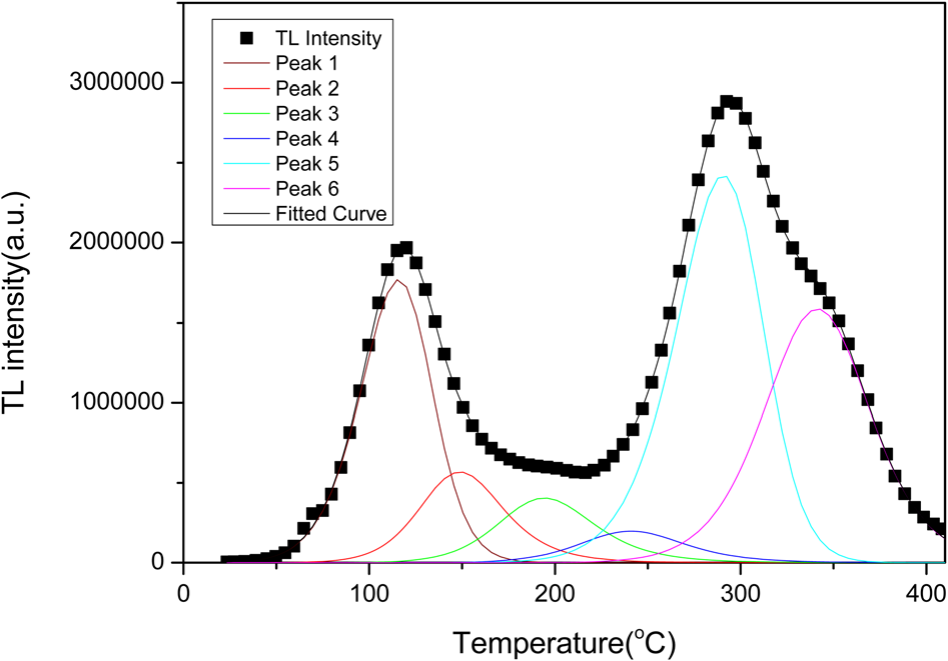
TL glow curve and fitted curve for LiMgPO_4_:Tm,Er.

Fig. 8 shows the TL glow curve obtained for LMP:Tm,Er powdered samples subjected to ^90^Sr beta radiation. The heating rate was 5 °C s^−1^. The shapes of the TL curves for the samples obtained using different irradiation times were basically consistent with those presented in Fig. 8. The TL glow curve of the LMP:Tm,Er phosphor exhibits 2 main TL glow peaks at approximately 120 °C and 300 °C in the range of 0–410 °C. The LMP:Tm,Er peak at approximately 120 °C is too low for use in practical TLDs. However, the main peak at 300 °C has good stability and can be used for routine TL dosimetric applications. For the Tm-doped LMP phosphor, the TL low-temperature peak is strong, and the high-temperature peak is weak.^60^ However, the high-temperature peak of LMP:Tm,Er is clearly more intense than the low-temperature peak, which indicates that the double-doped sample inhibits the shallow trap signal of the low-temperature peak and enhances the deep trap signal, which enhances the stability of the sample signal. Despite the nonradiative deexcitation of erbium ions, the sample exhibited high TL and OSL output.

The glow curve could not be fitted with a single kinetic peak and required at least 6 TL kinetic peaks to be fit (the trap activation energy is generally required to be in the range of 0.7–1.5 eV). These peaks correspond to trap centres formed at different depths. Fig. 8 shows the fitting results for the kinetic peaks, each of which can be described by the kinetic eqn (3):^61^3
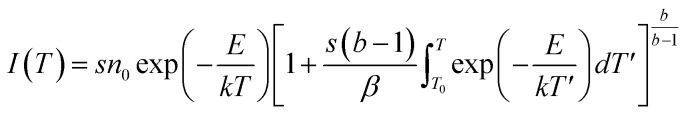
where *n*_0_ is the initial number of electrons captured in the sample; *E* is the activation energy for electron capture, eV; *s* is the frequency factor; *k* is the Boltzmann constant (0.862 × 10^−4^ eV K^−1^); *β* is the heating rate for the sample, K s^−1^; *T* is the absolute temperature in K; and *b* is the kinetic order. Fig. 8 shows that the dynamic TL peak is in good agreement with the experimental result. The fitting parameters of eqn (3) and the temperature (TM) for the kinetic peak are given in Table 2. The fitting results of the TL curve are as follows: the activation energy *E* is within 0.7–1.5 eV; the values of *b* for the kinetic order are 1–2; and the frequency factor *s* is 10^9^–10^11^.

**Table 2:** Fitted kinetic parameters for TL peaks

	Activation energy, *E*/eV	Frequency factor, s	Order of kinetics, *b*	Temperature of TL peak, TM/°C
Peak 1	0.77	2.7 × 10^9^	1.38	115
Peak 2	0.95	6.7 × 10^10^	2	148
Peak 3	1.01	2.0 × 10^10^	2	194
Peak 4	1.11	1.7 × 10^10^	1.92	241
Peak 5	1.32	1.4 × 10^11^	1.31	290
Peak 6	1.47	2.3 × 10^11^	1.69	341

Most CW-OSL luminescence models are based on mechanisms similar to TL. During the OSL process, charge transport is caused by photoexcitation from the capture level to the recombination centre. Thus, neglecting the probability that electrons in the conduction band are recaptured by traps, the simplest OSL luminescence model can be expressed as

4 *L*_OSL_ = *n*_0_*f*e^−*tf*^ = *L*_0_e^−*t*/*τ*^

where *f* is the excitation rate of electrons from the conduction band in the trap and *τ*= 1/*f* stands for the decay constant. The OSL corresponding to the simplest model is described by a first-order exponential decay function.

**Fig. 9:**
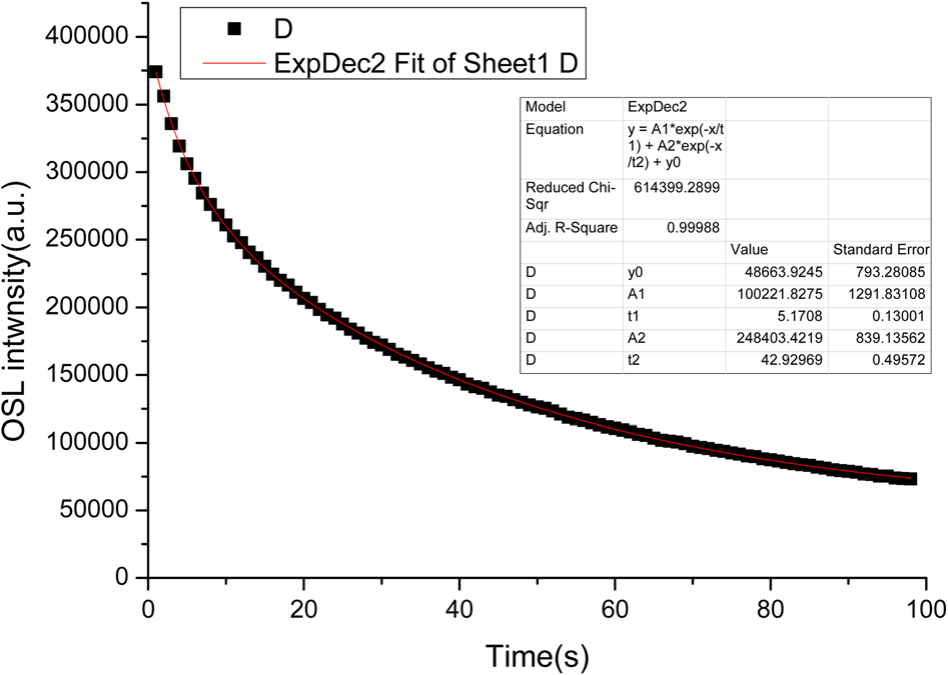
OSL decay curves of LiMgPO_4_:Tm,Er.

The LMP:Tm,Er phosphors were exposed to a ^90^Sr beta source. OSL measurements were conducted at room temperature. Fig. 9 presents the OSL decay curves of the LMP:Tm,Er phosphors. The decay curves were well fitted by a first-order exponential decay function. The OSL exhibited fast and slow decay components with corresponding decay constants of 5 s and 42 s.

### TL and OSL dose responses

**Fig. 10:**
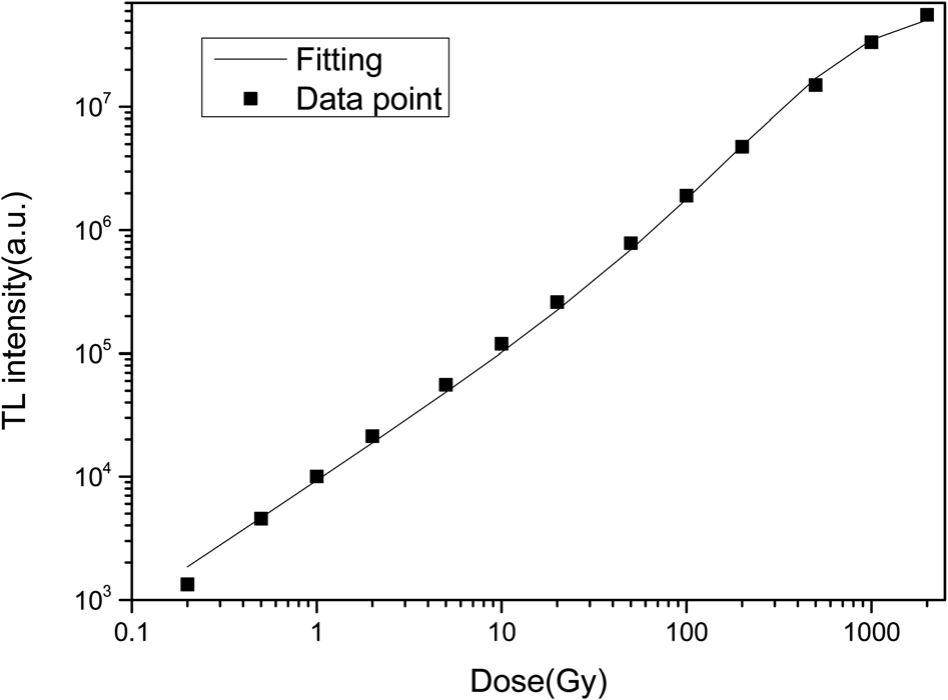
TL dose response of LiMgPO_4_:Tm,Er.

The TL dose responses of the LMP:Tm,Er phosphors were determined under exposure to a ^90^Sr beta source at doses ranging from 0.2 to 2000 Gy. Fig. 10 shows the TL dose response, where the area of the main TL peak is the ordinate and the irradiation dose is the abscissa. The curve was produced by using a composite response function proposed in the literature^62^ to fit the experimental data. The dose response *F*(*D*) satisfies the following equation:

5 *F*(*D*) = 1− e_0_^−*D*/*D*^ − (1 − *R*)(*D*/*D*_0_)e_0_^−*D*/*D*^

where *R* is a one-time action factor and *D*_0_ is the characteristic dose. The obtained nonlinear parameters of the TL dose response are *R* = 0.08 and *D*_0_ = 479 Gy. As 0 ≤ *R* ≤ 1/2, the LMP:Tm,Er sample dose response curve is linear-superlinear, which is mainly a secondary response.

**Fig. 11:**
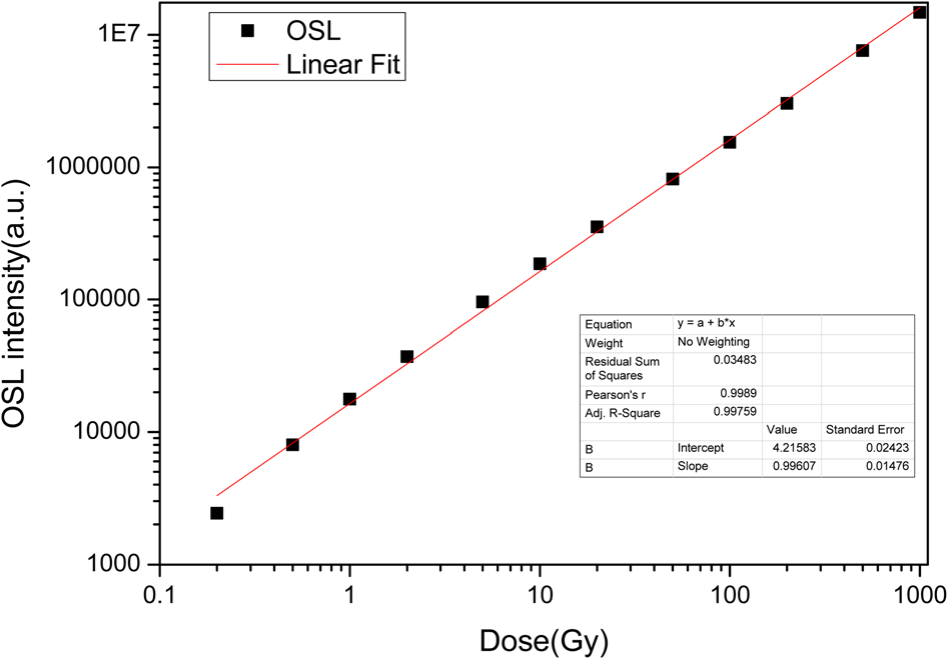
OSL dose response of LiMgPO_4_:Tm,Er.

The OSL dose responses of the LMP:Tm^3+^,Er^3+^ phosphors were determined under exposure to a ^90^Sr beta source at doses ranging from 0.2 Gy to 1000 Gy. The OSL signal was integrated from 0 s to 100 s. Fig. 11 indicates a linear OSL dose response of LMP:Tm^3+^,Er^3+^ to beta rays in the dose range of 0.2–1000 Gy.

### Reusability of LMP:Tm,Er

**Fig. 12:**
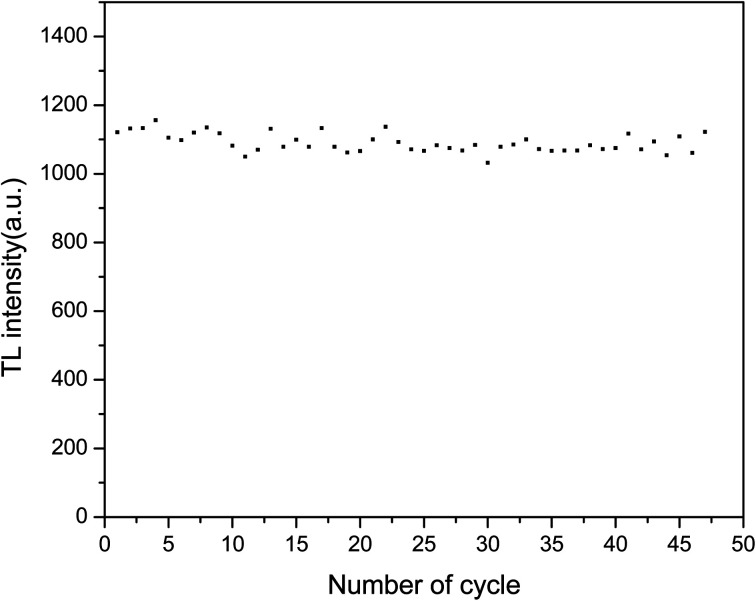
Repeatability of the TL signal of LiMgPO_4_:Tm,Er.

To investigate the reusability of the sample, the sample was repeatedly annealed, irradiated and changes in the TL intensity were measured. The sample was heated to 500 °C for 30 s, cooled to room temperature, irradiated with the same β ray dose, and then linearly heated from room temperature to 500 °C to obtain the TL glow curves. These steps were repeated 48 times. The area of the TL main luminescence peak obtained from each measurement was taken as the total number corresponding to this measurement, which is shown in Fig. 12. The relative standard error of 48 measurements was found to be 2.5%. The sensitivity of the LMP:Tm^3+^,Er^3+^ phosphor can be considered not to be significantly affected by irradiation and heating.

**Fig. 13:**
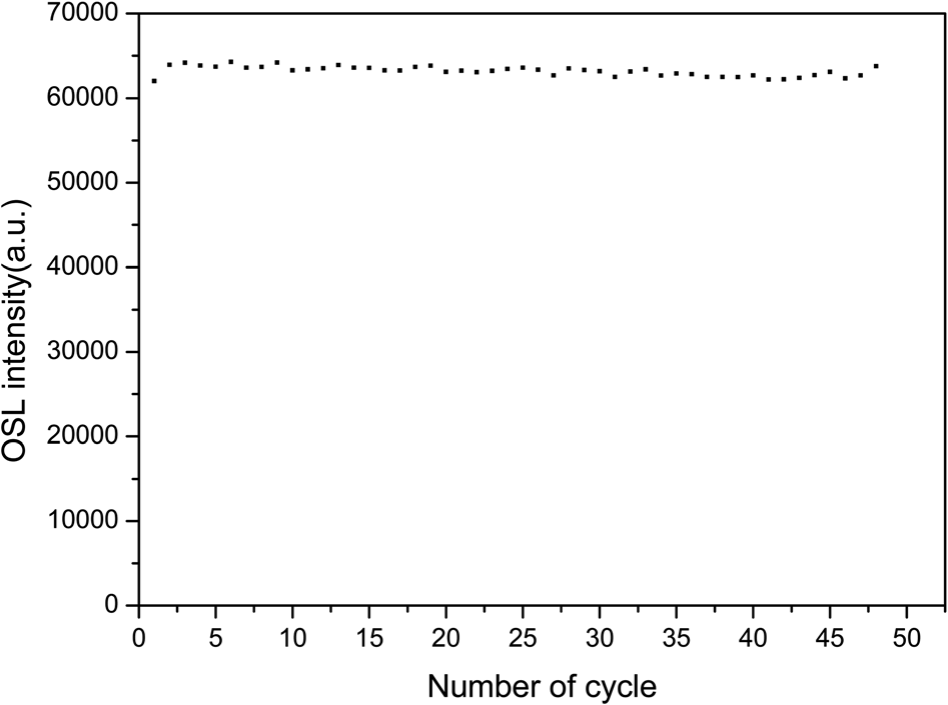
Repeatability of the OSL signal of LiMgPO_4_:Tm,Er.

To study the repeatability of the OSL signal of the phosphors, the samples were completely bleached at 500 °C for 300 s and exposed to the same ^90^Sr beta ray dose. The OSL decay curves were measured 48 times. The intensities of the OSL decay curve were summed to obtain the value reported in Fig. 13. There was little change in the sensitivity of LMP:Tm,Er under bleaching conditions. The relative standard deviation for 48 measurements was 0.9%. These results show that the TL and OSL signals of the LMP:Tm,Er phosphor have good repeatability.

## Conclusion

In this study, novel LiMgPO_4_:Tm,Er samples were synthesized by a high-temperature solid-state reaction. The TL and OSL under β-ray irradiation and XEOL of LiMgPO_4_:Tm,Er were studied in detail. DFT calculation results showed that the doping generation energy for Mg substituted by Tm/Er was smaller than that of Li substituted by Tm/Er at three doping concentrations. In the LiMgPO_4_-doped system, Mg was preferentially replaced by Tm/Er. The TL, OSL and TL3D spectra showed that LiMgPO_4_:Tm,Er emitted strong luminescence signals under different types of irradiation. The PL and TL3D spectra of the samples were dominated by the characteristic luminescence of Tm^3+^, and the luminescence caused by the 4f–4f transition of Er was not discernible in these spectra. Although Er^3+^ has no radiation excitation, Tm^3+^ has strong luminescence, such that the sample emitted strong TL and OSL signals. The luminescence peak temperature was consistent with that of the phosphate matrix, and two TL luminescence peaks appeared at 120 °C and 300 °C. The intensity of the high-temperature peak was significantly higher than that of the low-temperature peak, which enhanced the stability of the TL and OSL signals. The TL glow curve was fitted with six general TL kinetic peaks. The nonlinear parameters of the TL dose response were *R* = 0.08 and *D*_0_ = 479 Gy, and the OSL dose response was linear in the range of 0.2–1000 Gy. The TL and OSL signals of the LiMgPO_4_:Tm,Er phosphors had good repeatability. The OSL decay curve consisted of fast and slow decay components. The corresponding decay constants were 5 s and 42 s. Therefore, LiMgPO_4_:Tm,Er phosphors can be used for radiation dose measurement.

## Conflicts of interest

There are no conflicts to declare.

## Supplementary Material
